# Quantitative Modeling of GRK-Mediated β2AR Regulation

**DOI:** 10.1371/journal.pcbi.1000647

**Published:** 2010-01-22

**Authors:** Sharat J. Vayttaden, Jacqueline Friedman, Tuan M. Tran, Thomas C. Rich, Carmen W. Dessauer, Richard B. Clark

**Affiliations:** 1 Department of Integrative Biology and Pharmacology, University of Texas Health Science Center, Houston, Texas, United States of America; 2 Cell and Regulatory Biology Program, Graduate School of Biomedical Sciences, University of Texas Health Science Center, Houston, Texas, United States of America; 3 Department of Pharmacology, College of Medicine and Center for Lung Biology, University of South Alabama, Mobile, Alabama, United States of America; University of Houston, United States of America

## Abstract

We developed a unified model of the GRK-mediated β2 adrenergic receptor (β2AR) regulation that simultaneously accounts for six different biochemical measurements of the system obtained over a wide range of agonist concentrations. Using a single deterministic model we accounted for (1) GRK phosphorylation in response to various full and partial agonists; (2) dephosphorylation of the GRK site on the β2AR; (3) β2AR internalization; (4) recycling of the β2AR post isoproterenol treatment; (5) β2AR desensitization; and (6) β2AR resensitization. Simulations of our model show that plasma membrane dephosphorylation and recycling of the phosphorylated receptor are necessary to adequately account for the measured dephosphorylation kinetics. We further used the model to predict the consequences of (1) modifying rates such as GRK phosphorylation of the receptor, arrestin binding and dissociation from the receptor, and receptor dephosphorylation that should reflect effects of knockdowns and overexpressions of these components; and (2) varying concentration and frequency of agonist stimulation “seen” by the β2AR to better mimic hormonal, neurophysiological and pharmacological stimulations of the β2AR. Exploring the consequences of rapid pulsatile agonist stimulation, we found that although resensitization was rapid, the β2AR system retained the memory of the previous stimuli and desensitized faster and much more strongly in response to subsequent stimuli. The latent memory that we predict is due to slower membrane dephosphorylation, which allows for progressive accumulation of phosphorylated receptor on the surface. This primes the receptor for faster arrestin binding on subsequent agonist activation leading to a greater extent of desensitization. In summary, the model is unique in accounting for the behavior of the β2AR system across multiple types of biochemical measurements using a single set of experimentally constrained parameters. It also provides insight into how the signaling machinery can retain memory of prior stimulation long after near complete resensitization has been achieved.

## Author Summary

The β2 adrenergic receptor (β2AR) is involved in regulating many cellular processes such as smooth muscle relaxation in the airways and the vasculature. Drugs that activate the β2AR are used in treating asthma and chronic obstructive pulmonary disorder (COPD), and prolonged use of these drugs leads to the loss of their effects. Thus, a dynamic model of how the β2AR responds to different drugs is fundamental to their rational use. In this study a consensus model of G protein coupled receptor kinase (GRK)-mediated receptor regulation was formulated based on quantitative measures of six processes involved in β2AR regulation. This model was then used to simulate the consequences of manipulating key rates associated with the GRK-mediated β2AR regulation, leading to predictions which will provide a useful framework for further tests and elaborations of the model in basic and clinical research.

## Introduction

The β2 adrenergic receptor (β2AR) is intimately involved in the control of smooth muscle relaxation in airways and the vasculature, in stimulation of the heart, and numerous other physiologically important actions. Agonist stimulation of the β2AR causes activation of the Gs/cAMP/ protein kinase A (PKA) pathway. Agonist triggers β2AR desensitization that involves two pathways, G protein-dependent highly amplified PKA phosphorylation, and G protein-independent G protein coupled receptor kinase (GRK) phosphorylation that in turn triggers arrestin binding, internalization, recycling and resensitization [1]–[4]. Additionally, there is an important role for PKA regulation of adenylyl cyclase (AC) and phosphodiesterase activity in the desensitization of the response to β2AR stimulation [5],[6].

We recently examined the turnover profiles of cAMP in HEK 293 cells expressing only endogenous β2AR [6]. Our study of the β2AR desensitization was based on characterization of membrane localized cAMP in single cells with genetically encoded cyclic nucleotide-gated (CNG) channels in the presence of GRK and/or PDE inhibitors, and measurements of PDE activity. A model was developed that adequately described cAMP turnover based on GRK-mediated β2AR desensitization and PDE activation by PKA. Violin et al. [5] measured β2AR stimulation of cAMP profiles using a GFP-YFP-tagged EPAC sensor (ICUE2) following manipulation of GRK activities with siRNA to reduce levels of GRKs, and inhibitors to reduce PDE activity.

**Figure 1:**
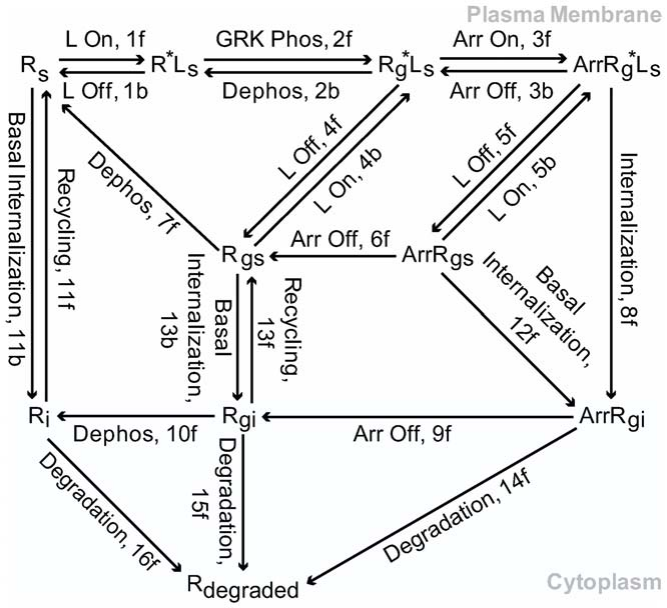
Reaction diagram of the GRK-mediated β2AR regulation. L is ligand; R^*^ is active state of β2AR; R_s_ and R_i_ are surface/plasma membrane and internalized β2AR; R_g_ is GRK-phosphorylated β2AR; Arr is arrestin. This reaction diagram describes the default model for simulations using the rate constants as described in Table 1.

As an extension of these studies, we have sought to model the GRK module of the β2AR desensitization and resensitization as it relates to measurements of phosphorylation, dephosphorylation, internalization, recycling, desensitization and resensitization (Figure 1). In previous studies we showed that HEK 293 cells overexpressing the β2AR was an excellent system for examining the biochemical parameters associated with the desensitization process. Importantly, the time course and extent of desensitization of the β2AR in these cells overexpressing the β2AR is similar to that of the endogenous receptor [7]. In the overexpression system, PKA-mediated phosphorylation of the β2AR shows an EC_50_ that is approximately 1000 fold lower than the EC_50_ for high occupancy-dependent GRK-mediated phosphorylation of the β2AR. Thus, it is possible to separate the GRK- and PKA-mediated desensitization of the β2AR into two different modules. At high concentrations of agonist we have shown that the GRK pathway accounts for almost all of the desensitization of the β2AR [8]. Here we have used a wide range of experimentally determined β2AR parameters (Table 1) to model and simulate the key steps in the consensus GRK pathway in response to varied agonist concentrations. The data simulated were obtained from over 90 distinct experiments performed in HEK 293 cells stably overexpressing the human β2AR. Accounting for all these disparate data using a single model provided strong constraints on the model, and ensured that its applicability was not restricted to only one class of data of the β2AR's response repertoire. The model provides a much needed quantitative description of the GRK pathway to β2AR desensitization and resensitization.

**Table 1:** Model parameters.

Reaction Name	Parameter (/min)	Reference/Rationale
Ligand (Agonist) On	k_1f_ = k_4b_ = k_5b_ = 500	Rates used to achieve fast ligand binding so that it is not rate limiting.^a^
Ligand (Agonist) Off	k_1b_ = k_4f_ = k_5f_ = 4	Rates used to achieve fast ligand binding so that it is not rate limiting.^a^
Ligand (Agonist) On – in the presence of an antagonist	k_1f_ = k_4b_ = k_5b_ = 0.005	Antagonist is assumed to behave as a competitive inhibitor [17] so the agonist binding rates are greatly reduced.
Ligand (Agonist) Off – in the presence of an antagonist	k_1b_ = k_4f_ = k_5f_ = 4	Agonist off-rates are unaffected in the presence of an antagonist that behaves like a competitive inhibitor.
GRK phosphorylation	k_2f_ = α[R*]1.4	Initial rate of GRK phosphorylation on treatment with 10 µM epinephrine = 0.7–1.4/min [19].^b^
GRK dephosphorylation	k_2b_ = k_7f_ = k_10f_ = 0.036	Phosphorylated receptor t_1/2_ = 18 min [16].
Arrestin On – to an agonist-bound receptor	k_3f_ = 27.0	Rate of arrestin binding = 26.6±5.9/min [10].
Arrestin Off – from an agonist-bound receptor	k_3b_ = 4.0	Rate of arrestin dissociation assumed to match measured K_d_.
Internalization	k_8f_ = 0.22	k_f_ = 0.22/min [19].
Basal Internalization	k_11b_ = k_12f_ = k_13b_ = 0.0085	Rates used to match negligible basal internalization [33].
Arrestin Off – from an agonist-free receptor	k_6f_ = k_9f_ = 11.0	Rate of arrestin dissociation = 10.8±1.2/min [10].
Receptor Degradation	k_14f_ = k_15f_ = k_16f_ = 0.004	t_1/2_∼3–4 hours [36].
Receptor Recycling	k_11f_ = k_13f_ = 0.09	k_f_ = 0.09/min [19].

a Off-rates for ISO ≥4/min [9], epinephrine >100/min [10]. For a K_d_ of 450 nM (epinephrine) and 283 nM (ISO) the calculated on-rates are very fast. Slowing down the forward-rates to 500/min does not affect the downstream events being simulated since they happen at a slower time scale. The ligand off-rate is not set at lower than 4/min in order to avoid making it rate limiting for arrestin dissociation.

b α = Coupling efficiency relative to epinephrine; ISO is assumed to have the same coupling efficiency as epinephrine since they are both full agonists. The relative coupling efficiencies for partial agonists are as follows, Epinephrine = ISO = 1, Fenoterol = 0.66, Formoterol = 0.63, Terbutaline = 0.33, Zinterol = 0.33, Albuterol = 0.25, Salmeterol = 0.13, Dobutamine = 0.04 and Ephedrine = 0.03. [R^*^] = ([R_total_] [Agonist]/([Agonist]+(K_d agonist_)); [R_total_] = 1; K_d epinephrine_ = 450 nM; K_d ISO_ = 283 nM. Simulated phosphorylation rate = α[R^*^]1.4 (for epinephrine); = α[R^*^]0.7 (for ISO).

## Results/Discussion

### Model of the GRK-Mediated β2AR Regulation Pathway

The present model of the GRK-mediated regulation of the β2AR pathway derives from consensus models that have appeared over the last decade [1]–[4] that includes activation, GRK phosphorylation, arrestin binding and dissociation, desensitization, internalization, recycling and resensitization of the receptor (see Figure 1). The experimental rate constants were derived from either new experiments or in some cases from previous publications as indicated (Table 1). The equations corresponding to these reactions (E1–E10) are described in Materials and Methods.

Our model incorporates several key assumptions. First, since all the parameters used for modeling of the GRK pathway were obtained using either isoproterenol (ISO) or epinephrine, full agonists for the β2AR, we assume a simple two state model for the receptor activation, where R_s_ indicates the inactive receptor on the surface of the plasma membrane and R^*^L_s_, the agonist-induced active receptor. Agonist binding to the receptor occurs with rapid on/off-rates. The off-rates for ISO have been estimated to be ≥4/min [9] and for epinephrine >100/min [10]. Based upon the K_d_s for epinephrine (450 nM) and ISO (283 nM), the on-rates were estimated to be very fast.

In the second set of assumptions we set the off-rates for a full agonist like epinephrine and ISO to be 4/min and chose an arbitrarily fast on-rate of ligand binding. This is done for the pragmatic purpose of running simulations without having to use the rapid rates, and also at the same time preventing the forward rates from being rate limiting for accessibility of the receptor for GRK phosphorylation. While the nuances of agonist binding to the β2AR and activation of Gs have been modeled in far greater detail [11],[12], for our purposes these specific interactions have been ignored because they occur at much shorter timescales compared to the 0.5 to 30 min events associated with GRK mediated β2AR desensitization and subsequent resensitization. We also assume that the off-rates for ligand from the receptor are independent of its status of phosphorylation or arrestin binding. The effects of these events on ligand off-rates have not been determined. There is evidence that arrestin stabilizes a high affinity state of ligand binding and therefore might reduce ligand off-rates [13]. Faster off-rates (5f in Figure 1) cause a faster resensitization following removal of agonist. Slower off-rates would lead to slower resensitization.

Third, we assume that the GRK phosphorylated β2AR (R_gs_, R_g_^*^L_s_) has reduced activity (0.7) compared to unphosphorylated β2AR, consistent with previous findings [14]–[16]. We also assume that any arrestin bound receptors in the plasma membrane are completely uncoupled. Additionally, to enable modeling of resensitization of β2AR stimulation of adenylyl cyclase following agonist treatment, and removal of the stimulus by addition of antagonist, we represented the antagonist like a competitive inhibitor that competes with the agonist for binding to the receptor leading to lower agonist binding rates [17]. Treating the antagonist as such necessitated the introduction of additional molecular species in the model, viz. agonist free GRK phosphorylated receptor, either bound to (ArrR_gs_) or free of arrestin (R_gs_). These molecules are usually at negligible levels, but at the instance of addition of an antagonist like propranolol or rapid washout of agonist, there is a transient increase in levels of these species. Finally, we ignore spontaneous activation of the β2AR, since in the absence of ISO treatment, GRK phosphorylation of the receptor is negligible [16], [18]–[20].

Fourth, we assumed that an activated β2AR undergoes a single event of GRK phosphorylation and that it can be dephosphorylated in the plasma membrane in addition to the cytosol. We set the rate of GRK phosphorylation to be dependent on [R^*^] which is the fraction of ligand-bound receptor. Thus in our model GRK phosphorylation is dependent on receptor activation and agonist concentration, which is consistent with agonist occupancy being the rate determining step for GRK phosphorylation for strong agonists [19], [21]–[23]. We previously identified three sites responsible for GRK phosphorylation of the β2AR, serines 355, 356, and 364 [8], and our conclusions have been supported by mass spectrometry [24]. All of our GRK phosphorylation studies are performed using an antibody that recognizes phosphorylation of serines 355, 356, but not of either alone. The experimentally measured GRK phosphorylation rates upon treating with epinephrine range from 0.7–1.4/min. We assume that since ISO is a full agonist, the phosphorylation rates fall within the same range. Intermediate phosphorylated states of other GPCRs like rhodopsin have been considered in kinetic models of shorter timescales [25]. For the longer timescales that we are modeling we ignore intermediary phosphorylated states and use the binding of our phosphosite antibody as readout of GRK phosphorylation of the receptor. The use of our anti-phosphosite antibody for kinetic studies has been validated by numerous groups [19], [26]–[29]. With regard to the cellular locale of β2AR dephosphorylation, our group has shown in several studies that the plasma membrane bound receptor, as well as that present in endosomes, can undergo dephosphorylation [16],[30].

Fifth, we assumed that the rate constants for arrestin binding to the receptor following agonist-induced GRK phosphorylation was 27/min and dissociation from an agonist-free receptor was 11/min [10] even though these were obtained from fluorescently tagged proteins. We estimated the arrestin dissociation rate from an agonist bound receptor complex (3b in Figure 1) to be ∼4.0/min given the stability of this complex from previous measurements of the K_d_ [31]. Arrestin binding in turn leads to clathrin coated pit internalization. Upon internalization of the β2AR we assume that there is rapid ligand dissociation consistent with the relatively high K_d_s and rapid on/off-rates of either ISO or epinephrine [9],[32]. We have shown that the rate of basal internalization is negligible [33] and have set the rate at a level that is 5% of the agonist-induced internalization.

Sixth, we assumed that the internalized β2AR can undergo; (i) dephosphorylation following arrestin dissociation [34],[35]; (ii) recycling with or without dephosphorylation [16]; and (iii) a very slow process of downregulation (t_1/2_ = 3–4 hours) [33],[36],[37]. Thus, in the present model that focuses on the rapid GRK-mediated events (0–30 min), we do not further discuss potential downregulation or de novo receptor synthesis as it contributes little to the events being modeled in these time scales. Also, in the model we allow any of the internalized receptors to be downregulated at the same low rate. At present, it is not clear what the precise mechanisms and pathways of downregulation of the β2AR involve, although we have shown that it is a biphasic process [37].

Finally, although GRK phosphorylation of the receptor and arrestin binding to the receptor are second order reactions, we represent them as pseudo-first order reactions. Our argument to do so is that both GRKs and arrestins are in excess of even the overexpressed receptor levels. Tran et al. [23] have shown that GRK2, 5 and 6 levels exceeded overexpressed β2AR levels by approximately 100 fold. Clark and Knoll [7] have shown that the extent of desensitization is similar with HEK 293 cells expressing endogenous (30 fmol/mg) versus overexpressed β2AR (3000 fmol/mg). This suggests that arrestin is at levels sufficient to give full desensitization even in a stable receptor overexpression system. Further, Menard et al. [38] have shown that HEK 293 cells have the highest relative levels of GRK and arrestin when compared to four other cell lines.

### ODE Model Captures the Behavior of the β2AR System

**Figure 2:**
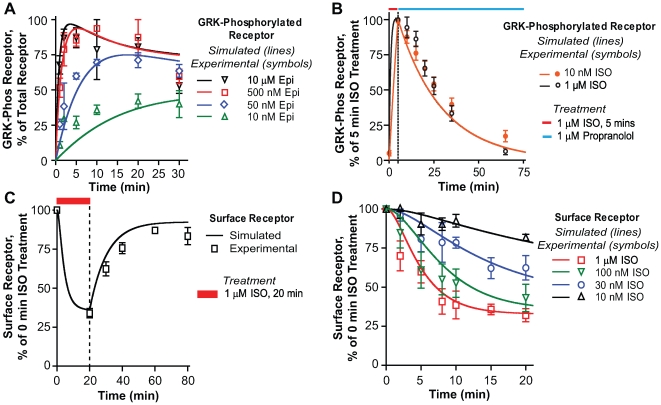
Comparisons of four experimental results with simulations of the model. Panels A–D: Comparisons of simulations (continuous lines) of the model shown in Figure 1, with experimental data obtained in HEK 293 cells stably overexpressing the FLAG WT β2AR (discrete data points). (A) Time course of GRK phosphorylation of the receptor on treatment with different concentrations of epinephrine [19]. (B) Dephosphorylation of the GRK phosphorylated site on the receptor after 5 min treatment with either 1.0 µM or 10 nM ISO (red bar) followed by addition of propranolol (blue bar) and measure of loss of GRK site phosphorylation. Phosphorylated receptor is expressed as a percent of phosphorylation achieved at the end of 5 min treatment with either agonist concentration [16]. (C) Recycling of the receptor after 20 min treatment with 1µM ISO followed by rapid washout of agonist [33]. (D) Internalization of the β2AR on treatment with various concentrations ISO as indicated. Surface receptor is measured by the loss of [^3^H]CGP-12177.

**Figure 3:**
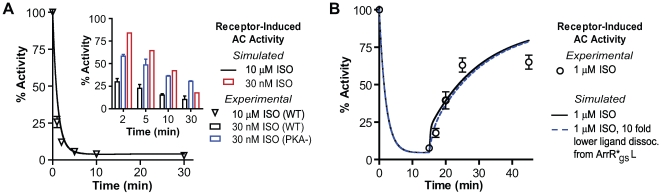
Validation of the model with two sets of experimental results. Validation of the simulations (continuous lines) of the model shown in Figure 1, with experimental data obtained in HEK 293 cells stably overexpressing the FLAG WT β2AR (discrete data points). (A) Desensitization of β2AR stimulation of adenylyl cyclase after treatment with 10 µM ISO. Inset shows desensitization obtained on 30 nM ISO stimulation. Red – simulated results; Black – WT cells; Blue - cells stably overexpressing β2AR lacking PKA phosphorylation sites (PKA-). At lower concentrations the model matches PKA- desensitization more closely since it does not include PKA-mediated β2AR desensitization. (B) Resensitization of the β2AR stimulation of adenylyl cyclase. WT β2AR were stimulated with 1µM ISO for 15 min, followed by addition of metoprolol as described in methods. Dotted line shows simulated % activity of the β2AR when ligand dissociation from arrestin-bound receptor complex is reduced by ten-fold.

Our mathematical model of GRK regulation of the β2AR was validated by running simulations and comparing these to *in vitro* experiments. The experiments include the six distinct biochemical measurements of the agonist-regulated WT β2AR desensitization and resensitization processes. These were derived from ∼90 distinct experimental measurements obtained across a range of ligand concentrations in HEK 293 cells expressing the WT β2AR (Figures 2, 3). The results of the modeling of these experiments are discussed below.

#### Phosphorylation

Figure 2A shows that the model captured the overall kinetics of the time course for GRK phosphorylation of the β2AR over a 1000 fold range of epinephrine stimulation from 0–30 min and in response to various partial agonists (Figure S1). GRK phosphorylation of the WT β2AR was measured in whole cell extracts with analysis of western blots using the phosphosite specific antibody, anti-pS (355, 356), in HEK 293 cells [19]. GRK phosphorylation of the β2AR was simulated by setting agonist concentrations and time of treatment equal to the corresponding experimental treatment. At the end of the simulation run, the sum total of the six GRK phosphorylated species, whether cytosolic or plasma membrane bound, was plotted against the experimentally measured GRK phosphorylated β2AR.

#### Dephosphorylation

Simulation of the dephosphorylation of the GRK phosphorylated β2AR is shown in Figure 2B. Dephosphorylation was measured after treatment of cells with either 1.0 µM or 10 nM ISO for 5 min followed by addition of 1.0 µM propranolol and measurement of the loss of WT β2AR phosphorylation using the anti-pS (355, 356) antibodies. Just like the experiments, the simulations were performed in two parts. The first part dealt with agonist treatment that allowed for phosphorylation of the receptor, and the second part involved treating with an antagonist which allowed for measuring/simulating dephosphorylation. The rate of agonist binding was calculated as described in Table 1, and the time and concentration of agonist treatment were dependent on the experimental protocol being simulated. At the end of the first run the concentrations of all forms of β2AR were set as initial concentrations for the second part. Antagonist binding was simulated as competitive inhibition. The net effect was that in the presence of an antagonist, agonist binding rates were reduced 100,000 fold, but the dissociation rates remain unchanged. At the end of the simulations the sum total of the six GRK phosphorylated species were plotted against the experimentally measured GRK phosphorylated β2AR. The model simulates well the dephosphorylation profile (Figure 2B) across a 100 fold agonist concentration range.

#### Internalization

Receptor internalization was measured using [^3^H]CGP-12177, following a 20 min treatment with ISO as previously described [16]. The internalization of the β2AR was simulated by setting agonist concentrations and the time of treatment same as the experimental protocol. At the end of the simulation run the sum total of all the plasma membrane bound β2AR was juxtaposed (Figure 2D) against the experimentally measured surface β2AR. Model simulations of βAR internalization (Figure 2D) matched the experimental results across a wide range of agonist concentrations.

#### Recycling

Receptor recycling was measured using [^3^H]CGP-12177 binding following treatment of cells with 1.0 µM of ISO and washout of agonist as previously described [8]. Recycling was simulated similar to dephosphorylation because both experiments involved a period of agonist treatment followed by either a rapid washout or antagonist treatment. At the end of simulating the recycling protocol the sum total of all the plasma membrane bound β2AR is juxtaposed (Figure 2C) against the experimentally measured surface β2AR. Visual inspection shows an excellent fit.

#### Desensitization

Phosphorylation, dephosphorylation, internalization, recycling and arrestin recruitment rates were explicitly modeled and the experimental measures of these rates defined the rates used in the model for these individual steps. Experimental measures of desensitization and resensitization were measures of downstream events, namely activation of AC. Since AC was not explicitly modeled, the receptor species capable of activating AC, namely, R_s_, R^*^L_s_, R_g_^*^L_s_ and R_gs_ were matched against the experimental measure of cAMP production, and in doing so we validated our model to data sets not used to develop the model.

The β2AR desensitization experiments (Figure 3A) were performed in HEK 293 cells stably overexpressing either WT β2AR or a β2AR lacking the two PKA consensus sites (S261, 262A and S345, 346A termed PKA-). The rationale for using the PKA- was to eliminate the contribution of PKA to the desensitization as previously described [8]. While the PKA component of desensitization is minimal at longer times of agonist treatment at high concentrations, it does contribute a small component of desensitization both at lower agonist concentrations and for shorter treatment times. Desensitization of β2AR stimulation of adenylyl cyclase was performed as previously described [19],[39]. The β2AR desensitization was simulated by setting agonist concentrations and time of treatment as per the experimental protocol being simulated. At the end of the simulation run, the total of the three active forms of the β2AR and naïve receptor was juxtaposed (Figure 3A) against the experimentally measured active β2AR. As expected from the contribution of the PKA desensitization, we found that our GRK-mediated β2AR regulation model underestimated the experimentally determined desensitization over the first two minutes of agonist treatment for the WTβ2AR, but there was good agreement with longer treatment times. However, our model matches well the desensitization measured in the PKA- cells that overexpress the β2AR lacking the PKA consensus site, thus, supporting our argument that the model misses the PKA component that contributes to immediate early desensitization.

#### Resensitization

Figure 3B shows the results of simulation of receptor resensitization. Resensitization of β2AR stimulation of AC following agonist treatment (15 min, 1.0 µM ISO) and addition of the low affinity antagonist, metoprolol for the times indicated was measured as described in Materials and Methods. The resensitization of the β2AR was simulated in two parts, as described for dephosphorylation and recycling above. At the end of the simulation run the sum total of all the active forms of the β2AR is juxtaposed (Figure 3B) against the experimentally measured active β2AR. We see that using the default model there is a spike in the initial rate of resensitization relative to the experimental values. The spike can be ablated if we assume a ten-fold lower rate of ligand dissociation from an arrestin-bound receptor complex based on the apparent stability of this complex [31].

#### Sensitivity analysis of desensitization and resensitization

The univariate sensitivity analyses were carried out to test the effect of up to a twenty-fold increase or decrease in individual rates on the simulation results of desensitization and resensitization under conditions described in Figure 3 (Figure S2, S3).

Significant deviations from experimental desensitization measurements were obtained for perturbations of only three rates. Consistent with the model, decreasing GRK phosphorylation (k_2f_) reduced the desensitization at earlier time points. Reducing arrestin affinity for the receptor-ligand complex (k_3f_, k_3b_) decreased desensitization at later time points.

The resensitization sensitivity analysis (Figure S3) results are complicated because we first desensitize the system for 15 min. This affects the starting values of resensitization. Decreasing internalization of ArrR_g_^*^L_s_ (k_8f_) reduces the amount of receptor desensitization achieved at 15 min (see conditions in Figure 3B). Thus at the start of resensitization simulation we begin at a higher baseline and this overestimates the resensitization. As the simulation progresses there is no significant effect of perturbing internalization rates. Increasing recycling (k_11f_, k_13f_) reduces the amount of internalized receptor and therefore leads to an overestimation of resensitization.

### Model Predictions for Variations in Rates of GRK Phosphorylation and Arrestin Binding

In various cell types GRKs and arrestins differ in overall expression levels, localization [40],[41], post-translational modifications [42]–[45] and have been frequently targeted for knockdown or overexpression [28], [44], [46]–[48]. We were interested in seeing model predictions when we varied the levels or activity of these two β2AR regulatory proteins on phosphorylation, desensitization and internalization over a 0–30 min time course. To simulate this we varied GRK phosphorylation and arrestin binding rates ten-fold, both above and below the default rates.

#### GRK rates

**Figure 4:**
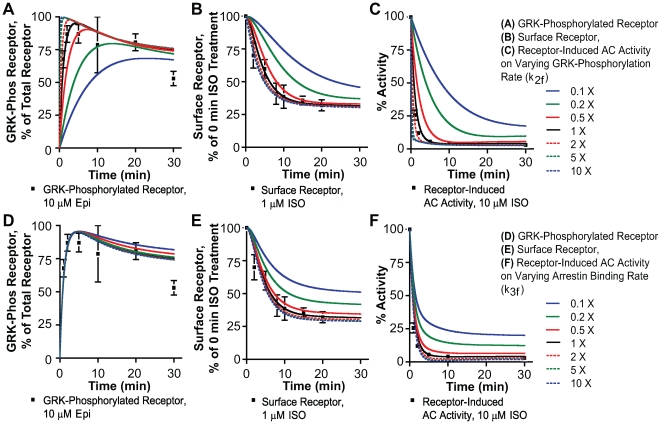
Simulated effects of varying rates of GRK phosphorylation and arrestin binding. A–C: Simulated effects of ten-fold variation in GRK phosphorylation rates on (A) phosphorylation, (B) internalization and (C) desensitization. D–E: Simulated effects of ten-fold variation in arrestin binding rates on (D) phosphorylation, (E) internalization and (F) desensitization. Experimental data as given in Figure 2A, C, and Figure 3B.

Decreasing the GRK phosphorylation rates (Figure 4 A–C), at saturating concentrations of ISO, resulted in decreased initial phosphorylation levels, whereas the steady state rates were unchanged (Figure 4A). Increasing GRK phosphorylation rates did not have a marked effect on the initial phosphorylation and maximum phosphorylation. This is an interesting observation because GRK knockdown and overexpression studies are often performed at steady state and at saturating agonist concentrations. In principle this increases the risk of false negative results. Our data and simulations suggest that a more rigorous approach is measurement of initial rates of β2AR phosphorylation at subsaturating agonist concentrations. We show that the simulated effects of variations in rates of GRK phosphorylation are more pronounced following 50 nM ISO treatment (Figure S4). With respect to internalization (Figure 4B), lowering the GRK phosphorylation rates has marked effects on the initial rates of internalization, with little effect on the maximum amplitude. The effects on desensitization due to variation in phosphorylation rates were similar to the effects on phosphorylation, but phase shifted because these are sequential events (Figure 4C). As with phosphorylation therefore, measurements ideally should be made at the earlier time points and low agonist concentrations to detect significant changes.

#### Arrestin rates

Similar to the analyses above, we varied the rates of arrestin binding by ten-fold and studied the effect it had on phosphorylation, desensitization and internalization (Figure 4D–F). Neither increasing nor decreasing arrestin binding rates had significant effects on the initial maximum amplitude of phosphorylation (Figure 4D). Reduction in arrestin binding rates (Figure 4E) reduced the extent of internalization (∼40% between 5–10 min), but increasing the rates did not increase the maximum internalization any further. The most drastic effect of varying arrestin binding rates was on the extent of receptor desensitization (Figure 4F). Here again, like internalization, a marked effect was visible only on reducing the arrestin binding rates and not on increasing the rates.

### Phosphatase Access to the Membrane and Phosphorylated Receptor Trafficking are Required to Account for β2AR Response Characteristics

There has been some controversy about the possibility of plasma membrane dephosphorylation of the GRK phosphorylated receptor and the recycling of the phosphorylated receptor [30], [34], [49]–[51]. Recently we have shown that (i) β2AR can be dephosphorylated when internalization is blocked with either hypertonic sucrose treatment or through the use of a dominant negative form of dynamin [30], and (ii) that dephosphorylation occurs with no detectable internalization [16]. We have determined surface dephosphorylation rates of ∼0.04/min through two different experimental methodologies (data not shown) as follows; (i) dephosphorylation was measured after 30 s treatment with 1 µM ISO at which point there shouldn't be significant internalization; or (ii) after pretreatment of cells with concanavalin A which reduced internalization by ∼80%.

**Figure 5:**
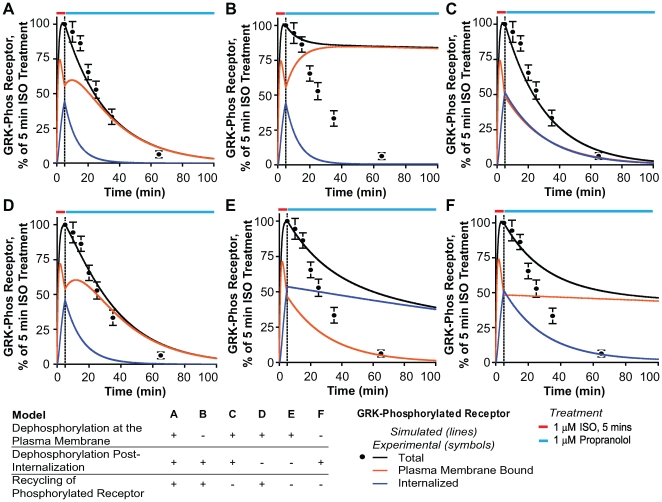
Simulated effects of phosphatase location and recycling of phosphorylated β2AR on receptor dephosphorylation. HEK 293 cells stably overexpressing WT β2AR were treated for 5 min with 1 µM ISO (red bar) followed by washout and addition of 1 µM propranolol (blue bar). Experimental data [16] are shown as discrete points with standard errors and the simulations are shown as continuous lines. The black lines are the total phosphorylated receptor, red indicates the phosphorylated receptor on the plasma membrane and blue indicate internalized levels of phosphorylated β2AR. (A) Model A allows for dephosphorylation of both the internalized and plasma membrane bound receptor along with recycling of phosphorylated and dephosphorylated receptor. (B) Model B disallows plasma membrane dephosphorylation but allows both dephosphorylation of the internalized receptor and recycling of phosphorylated receptor. (C) Model C allows for dephosphorylation of both the internalized and plasma membrane bound receptor but limits recycling to only dephosphorylated receptor. (D) Model D allows for dephosphorylation only at the plasma membrane and also allows recycling of phosphorylated receptor. (E) Model E allows for dephosphorylation only at the plasma membrane and disallows recycling of phosphorylated receptor. (F) Model F allows for dephosphorylation only after internalization and prevents recycling of phosphorylated receptor.

Plasma membrane dephosphorylation has also been demonstrated for other GPCRs such as the D1 dopamine receptor [52], and for the TRH receptor [53]. To further investigate these processes, we created six different models (Figure 5A–F) in which the effect of plasma membrane and internalized β2AR dephosphorylation and recycling of phosphorylated receptor on total dephosphorylation was explored in six possible combinations.

All six models were tested (Figure 5A–F) with various combinations of plasma membrane and cytosolic dephosphorylation and recycling of phosphorylated receptor. To aid in our interpretation of the cellular distribution of the phosphorylated β2AR, we plotted the total (black), the surface (red) and cytosolic (blue) phosphorylated β2AR. Of the six models tested only three (Figure 5A, C, D) matched the experimentally determined dephosphorylation kinetics. Figure 5A shows results from model A, the default model (Figure 1, Table 1) used throughout the paper that matches other biochemical readouts presented (Figures 2, 3).

In Figure 5C, disallowing recycling of phosphorylated receptor does not affect the simulated total rate of β2AR dephosphorylation. Even though this scenario is theoretically tractable, it does not match experimentally observed phenomena. Tran et al. [16] showed through immunolocalization of pS (355,356) on β2ARs that the phosphorylated receptor can recycle back to the plasma membrane after 3 min of recycling after a 5 min treatment with 1µM isoproterenol. Pippig et al. [34] have shown that treating A431 cells with a phosphatase inhibitor did not affect the recycling of β2ARs in these cells. This would mean that phosphorylated receptor would have the same rate of recycling as the net rate of recycling. In Figure S5A–E we test model C against other biochemical readouts and show that in the absence of phosphorylated receptor recycling, the model does not match well with internalization (Figure S5B), recycling (Figure S5C) and resensitization (Figure S5E).

Figure 5D explores a scenario that allows for dephosphorylation only at the plasma membrane and allows for recycling of the phosphorylated receptor. The measured dephosphorylation rates match the simulated dephosphorylation quite well. Though theoretically feasible, absence of cytosolic dephosphorylation is at variance from experimental observations of the β2AR system [16],[34],[35]. In Figure S5F–J we test this model for other biochemical measurements and show that in the absence of cytoplasmic dephosphorylation, plasma membrane dephosphorylation has to be 30 fold higher to match the phosphorylation data (Figure S5F).

In Figure 5E we test a scenario that allows for dephosphorylation only at the plasma membrane and disallows recycling of phosphorylated receptor. In this paradigm the total dephosphorylation rates are markedly reduced. In Figure 5F, which models the receptor system as per the current dogma [22],[54], we show that in the absence of both plasma membrane dephosphorylation and recycling of phosphorylated receptor, the system fails to achieve more than 50% dephosphorylation which is at odds with our experimental data. The major consequence of this model is that it essentially freezes the plasma membrane level of phosphorylated β2AR. This phenomenon is exaggerated when recycling of phosphorylated receptor is allowed as in Figure 5B. Thus of the three models that capture dephosphorylation kinetics of the receptor, only one model viz. model A (Figure 1, Table 1, Figure 5A) can account for six different types of biochemical readouts (Figures 2,3).

### Frequency Coding in the β2AR Signaling System

In an earlier study of the resensitization of β2AR stimulation following agonist treatment, we found that there was a rapid phase of resensitization that we attributed to the rapid dissociation of arrestin and a slower phase corresponding to recycling of receptor (see Figure 3B) [16]. The rapid phase occurred with minimal dephosphorylation, clearly dissociating it from resensitization. We and others [10],[19] speculated that the slow rate of dephosphorylation would lead to a “memory of desensitization” upon a washout phase followed by second treatment with agonist.

These, as well as most previous studies, were performed with saturating levels of agonist for extended periods of time. *In vivo* this type of exposure likely rarely occurs. Rather, the β2AR is exposed to different amplitudes and frequencies of agonists in different tissues; e.g., β2AR in the synapse is exposed to a higher concentration of norepinephrine, but the delivery is pulsatile [55],[56] due to the rapid removal and reuptake of norepinephrine, whereas following release of epinephrine from the adrenal gland, much lower levels of agonist concentrations in the bloodstream are achieved for relatively longer periods.

**Figure 6:**
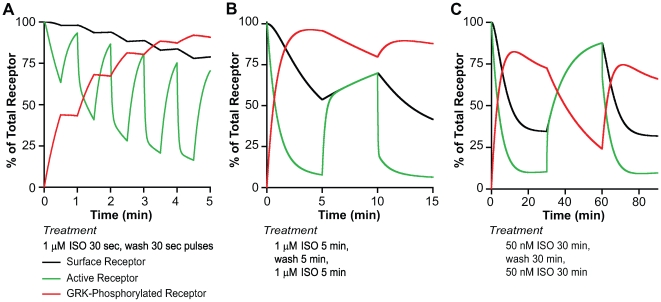
Simulations of the effects of frequency modulation. In these panels we describe the effect of varying the frequency of stimulation on surface (black), phosphorylated (red) and active (green) receptor species. (A) Rapid stimulation with a train of 1 µM ISO pulses for 0.5 min followed by a 0.5 min immediate washout. Note that this achieves more than 80% desensitization with only 20% internalization. (B) This panels shows the results of a 5 min stimulation with 1.0 µM ISO and a 5 min washout. (C) Simulation of a prolonged 30 min stimulation with 50 nM ISO followed by washout of 30 min. This panel shows that even with low β2AR occupancy (15%) the prolonged stimulation time gives substantial desensitization.

To explore the predictions of our model under various frequencies of agonist stimulation, we first simulated a scenario where there is a rapid burst of a high concentration of agonist for 30 s duration followed by 30 s washout. We assumed instantaneous agonist dissociation from the receptor (Figure 6A). We observed that under this stimulation paradigm close to 80% desensitization (green – active receptor) is achieved with almost 100% GRK phosphorylation (red – phosphorylated receptor) and only about 20% internalization (black – surface receptor). Following removal of agonist, resensitization is rapid due to rapid arrestin dissociation. In spite of this near complete recovery, we show that during this pulsatile activation of the receptor, the receptor “remembers” prior exposure to an agonist and desensitizes much more strongly on subsequent exposures. This is because of the accumulation of the phosphorylated receptor due to slower dephosphorylation.

In Figure 6B we stimulate with the same concentration of agonist (1µM ISO) as used in the previous figure but instead of rapid pulsatile delivery we model a continuous delivery for 5 min followed by a wash for 5 min and subsequent restimulation for 5 min. Under this paradigm almost a 95% desensitization is achieved but with more than 50% internalization. In Figure 6C we test how the system would behave when challenged with sub-saturating levels of agonist for longer periods of time. We simulated a β2AR response to 50 nM ISO for 30 min followed by 30 min wash and restimulation. Under longer periods of agonist treatment (Figure 6B, C) the resensitization is biphasic (see Figure 3B). The rapid phase of resensitization is dependent upon arrestin dissociation while the slower phase is dependent upon recycling and dephosphorylation [16].

The latent memory that we described in Figure 6A is only observed at higher frequencies of agonist stimulation. For periods of longer stimulation (Figure 6C), as might represent treatment with a strong stable agonist in diseases such as asthma, receptor recycling plays an important role in resensitization. Surprisingly, if the longer term treatment is with a weak agonist like albuterol, a very strong desensitization occurs since this agonist at saturation is equivalent to the 50 nM treatment with ISO as previously discussed [57]. The consequences of weak partial agonists are shown in the supplementary Figure S1A, B.

### Slow Dephosphorylation and Rapid Arrestin Dissociation Account for a Latent Memory of β2AR Response to Agonists

**Figure 7:**
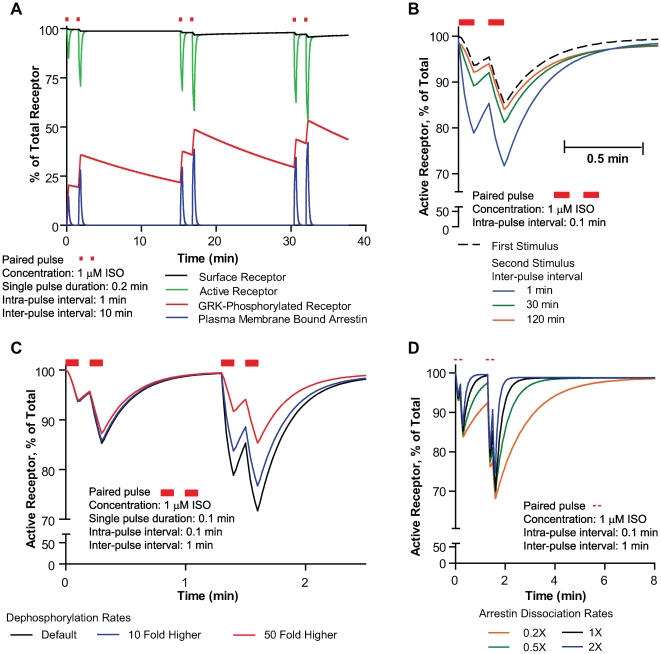
Basis for “Cellular Memory” in the β2AR signaling machinery. (A) Simulation of activation of β2AR by paired pulses of 1 µM ISO. Higher desensitization is obtained for the second and third pulse. Colors indicate simulated receptor species as indicated in the figure. (B) Decay in memory of prior stimuli on increase in inter-pulse period from 1–120 min. (C) Effect of up to 50 fold increase in surface dephosphorylation rates on memory of prior stimuli. Default dephosphorylation rate is 0.036/min. (D) Effect of arrestin-β2AR complex stability on desensitization time courses simulated by varying arrestin dissociation rates from the ligand-free complex on the surface. Default arrestin surface dissociation rate from the ligand-free complex is 10/min.

Having shown that the β2AR signaling system could “remember” prior stimuli (Figure 6A) we wanted to explore the inter-pulse time (time between paired pulse stimulation) dependency and effects of varied receptor dephosphorylation and arrestin dissociation on this latent memory. In Figure 7A we simulate a paired pulse paradigm of receptor activation with 1µM ISO. As we predicted, even though resensitization is rapid, the β2AR system retained the memory of the previous paired pulse stimuli following restimulation and desensitized more strongly in response to subsequent stimuli as shown by the plot of “active” β2AR (green lines). The simulations show that this latent memory is due to the progressive accumulation of phosphorylated receptor on the surface (red). This primes the receptor for faster arrestin binding to surface receptor (blue) on subsequent agonist activation leading to a greater extent of desensitization. Note that there is no significant internalization following the pulsatile stimuli (black lines – surface receptor). Progressively increasing the inter-pulse time reduces the memory (Figure 7B), as it allows significant dephosphorylation to occur. The memory is robust and survives beyond 30 min after the first stimulus. When the dephosphorylation rates are increased 2–50 fold over the default rate we see that a 50 fold increase is required to ablate the memory of the previous 6 s pulse of agonist (Figure 7C). Going in the other direction, the result of decreasing the dephosphorylation rate 50 fold would result in a profound locking-in of latent memory (simulation not shown).

Some GPCRs have an apparently much higher affinity for arrestin such that arrestin does not dissociate rapidly following internalization [58]. To investigate this possibility we increased the affinity of arrestin for the receptor agonist complex in the model by decreasing the dissociation rate of arrestin from the complex. Increased residence of arrestin with the receptor complex increased the time required for resensitization (Figure 7D), whereas decreasing the residence time of arrestin on the receptor allowed for rapid resensitization. We also increased the stability of ligand binding to the receptor/arrestin complex by decreasing the off-rate 100 fold, and still the property of memory was not lost (Figure S6), although recovery time is extended. To summarize, the latent memory that we predict in the β2AR system is due to the slow dephosphorylation and rapid arrestin dissociation from the receptor.

### Partial Agonism

Another application of the model is for predictions concerning partial agonists. For most partial agonists the initial rate of ligand-induced GRK site phosphorylation is proportional to coupling efficiency (with the notable exception of cyclopentylbutanephrine) [18],[19],[59]. In our simulations of partial agonist phosphorylation (Figure S1A, B) we are able to predict the extent and time course of phosphorylation solely by setting agonist occupancy proportional to coupling efficiency where the coupling efficiency of epinephrine, a full agonist, was set at 1.

Prolonged treatment with saturating concentrations of salmeterol, a clinically important drug for asthma therapy, results in decreased β2AR internalization relative to ISO, even though the GRK phosphorylation of the β2AR is of comparable levels (Figure S1A) [19],[57],[59]. Overexpression of arrestin restores some internalization [57]. This has led us to propose that salmeterol stabilizes an altered state of the receptor that has less affinity for arrestin which leads to a decrease in β2AR internalization. We modeled (Figure S1C) β2AR internalization on treatment with salmeterol and found that a simulation based only on coupling efficiencies overestimates grossly the extent of internalization. This discrepancy can be corrected in part if we reduce by ten-fold the rate of arrestin binding to a salmeterol-bound receptor complex. However it takes a ten-fold decrease in the rate of arrestin binding and a ten-fold increase in rate of arrestin dissociation from a salmeterol-bound receptor complex to match the negligible amount of experimentally measured internalization. This is congruent with our idea that salmeterol-bound GRK-phosphorylated β2AR complex (ArrR_g_^*^L_s_) has a lower affinity for arrestin.

Attempts at measuring salmeterol-induced desensitization have been frustrated by the inability to wash salmeterol out. However our previous work suggested much less desensitization relative to a full agonist [57], [59]–[61]. Now it is possible for us to simulate salmeterol-induced desensitization using a model that matches other experimental measurements like salmeterol-induced β2AR phosphorylation and internalization. The result of the simulation (Figure S1D) clearly supports the contention that salmeterol causes a much reduced desensitization relative to ISO when both the on- and off-rates of arrestin are changed ten-fold as in Figure S1C. This may also help explain why salmeterol, a weak partial agonist, is an effective treatment for asthma.

### Model Limitations

Despite the ability of the model to capture the broad range of data shown, a few limitations exist. The model in its current form does not include adenylyl cyclase, PDE, or PKA-mediated phosphorylation of the receptor. In the absence of the PKA reactions the model cannot capture the behavior of the β2AR signaling system at low agonist concentrations or PKA induced PDE regulation. We previously showed that prestimulation of PKA phosphorylation with forskolin does not alter the time course of GRK phosphorylation [19], however, at high concentrations of agonist the GRK phosphorylation appears to reduce PKA site phosphorylation [20]. A further complication concerns the possibility of AC regulation. Since we deal with only receptor-level desensitization we have ignored these downstream regulatory events.

We suggest a form of “cellular memory” occurs that allows the system to remember prior stimuli which are difficult to measure experimentally. For intact cell analysis this is confounded by the rapid hydrolysis of cAMP caused by PKA stimulation of PDE and the consequent difficulties in interpretation even with saturating levels of PDE inhibitors. Resensitization experiments need to be carried out in the presence and absence of internalization/recycling to show that under certain patterns of stimuli the system can show a greater desensitization to the second stimuli as suggested by the simulations. We also need to show that this effect is abrogated by addition or upregulation of phosphatase activity. Our simulations indicate that this potential upregulation must be extreme (Figure 7C) to significantly change the retention of memory. In that regard we recently reported on mutations of 3 lysines that greatly reduce ubiquitylation, and surprisingly cause an ∼5–7 fold increase in the rate of dephosphorylation of the β2AR [36]. Consistent with our model this increase did not appreciably alter either desensitization or internalization of the receptor.

We show that the currently held dogma that disallows the possibility of plasma membrane dephosphorylation of the receptor and recycling of the phosphorylated receptor is clearly incompatible with our modeling and previous experimental results. One caveat though is that the effect of ligand-independent internalization of the β2AR on dephosphorylation kinetics has not been explored in detail. Ligand-independent internalization has been shown to occur in other GPCRs like the cannabinoid CB1 receptor [62] and melanocortin MC4 receptor [63]. We have previously shown in our cell lines that in the absence of agonist there is no measureable internalization of the β2AR [33]. Recently [64] it was shown that in HeLa cells transiently transfected with β2AR or M3R there was measureable amounts of ligand-independent internalization. It remains to be further examined if this was an artifact of transient transfections or if constitutive internalization of β2AR does occur in other cell lines and has an effect on dephosphorylation kinetics. Preliminary simulations (data not shown) on the effect of ligand-independent internalization on dephosphorylation kinetics in model F (from Figure 5) show that an increased rate of constitutive internalization will not rescue the poor behavior of model F.

### Concluding Remarks

Our long-term goal is to develop comprehensive, quantitative models of β2AR-mediated signaling pathways. Such quantitative models serve as a summary of the current state of understanding in the field, and have many important applications: (i) understanding of the mechanisms underlying the differences between β2AR response in different tissue types; (ii) simulation of clinically observed tachyphylaxis in β2AR associated with prolonged use of β2AR agonists in asthma and COPD treatment; (iii) hypothesis testing and generation of experimental predictions. The present model represents a significant advance toward this goal, since it is able to account for many salient β2AR response features in various experimental studies performed in different cell lines.

## Materials and Methods

### Experimental Methods

For the majority of the new experiments performed for this work, we used HEK 293 cells stably overexpressing the WT β2AR tagged with an N-terminal FLAG epitope [16]. For select experiments we used a WT β2AR tagged with HA on the N-terminus [33], or WT β2AR tagged with both an HA (N-terminus) and His6 (C-terminus) – Hβ2ARH [39],[59]. All of the stable HEK 293 lines were used with β2AR levels of from 3–6 pmoles/mg membrane protein. We have found that these different tags do not significantly alter any of the desensitization parameters discussed in Table 1 [16],[20],[30],[33],[39],[59], therefore for the present discussion we refer to these stably expressing cell lines as expressing WT β2AR. Also in some previously published experiments we used epinephrine instead of ISO, and again we have found no differences in the effect these strong agonists have on the desensitization parameters at similar levels of receptor occupancy [19].

#### β2AR internalization

WT βAR were treated with ISO for varied periods of time. Following agonist treatment and extensive washing, β2AR internalization was measured on intact cells using the binding of [^3^H]CGP-12177 (20 nM) as previously described [16]. [^3^H]CGP-12177 is a hydrophilic antagonist that labels only surface (plasma membrane) receptor at 0–4°C. The binding assay is conducted ±1µM alprenolol, a β2AR antagonist that is used to determine non-specific binding of [^3^H]CGP-12177. The measure of surface receptor at time 0 is assumed as 100% surface receptor.

#### β2AR recycling

WT β2AR were treated with agonist (ISO) for 20 min to generate maximal internalization. Following agonist treatment the cells were washed extensively to remove agonist, incubated for the times indicated at 37°C to allow recycling. Recycled β2AR levels were determined with [^3^H]CGP-12177 [8]. The measure of surface receptor at time 0 is assumed as 100% surface receptor.

#### β2AR desensitization and resensitization

Desensitization of β2AR stimulation of adenylyl cyclase was measured as previously described [8],[16]. Briefly either the WT β2AR, or for the experiment shown in Figure 2B, cells stably expressing the β2AR with the two PKA consensus sites substituted with alanines [8] were incubated with agonist (ISO) at various concentrations and after washout of agonist, membranes were prepared on sucrose step gradients. The extent of desensitization was measured by determination of the increase in EC_50_ for ISO stimulation of adenylyl cyclase relative to controls and the results expressed as fraction activity remaining as discussed [16]. There is also a 35% decrease in V_max_ that our previous studies have shown is from downstream effects, most likely on AC [8],[65]. So in our current model of receptor-level desensitization we ignore these V_max_ changes.

For resensitization of WT β2AR cells, propranolol cannot be used for the blockade of agonist stimulation, since its rate of dissociation is too slow to allow for a time course measurement of resensitization. To circumvent this problem we used 100 µM metoprolol [16], a low affinity (240 nM K_d_) antagonist of the βARs. After the various times allowing resensitization, cells were washed free of metoprolol, and membranes were prepared and assayed for ISO stimulation of adenylyl cyclase as previously detailed [16]. The measure of AC activity in membranes at time 0 is assumed as 100% activity in desensitization and resensitization experiments.

#### β2AR phosphorylation and dephosphorylation

GRK phosphorylation of the β2AR was determined using anti-phosphosite-specific antibodies to residues S(355, 356) as described previously [19]. Briefly, WT β2AR were incubated with ISO for varying times after which the whole cell β2AR was extracted with solubilization buffer. The extract was incubated with PNGase to remove glycosyl residues, treated with SDS sample buffer and run on PAGE. After transfer the levels of GRK site phosphorylation were determined by western blots. Phosphorylation data was first normalized to receptor levels and then for comparison between experiments to the maximum epinephrine-stimulated value. For dephosphorylation WT β2AR were treated for 5 min with either 1.0 µM or 10 nM ISO, after which the cells were washed and incubated with medium containing 0.1 µM propranolol to block further ISO stimulation for the times indicated [16]. The loss of GRK site phosphorylation was then measured by western blots as discussed above. In dephosphorylation experiments the measure of phosphorylation at 5 min was set as 100% phosphorylation for different agonist concentrations.

### Computational Methods

#### Simulation methodology and analysis tools

Since no single experiment gives the full range of data needed to adequately constrain the model we used an amalgamation of over 90 different data points from distinct experiments from three different cell types. The data sets chosen for modeling spanned measurements across every possible step in the signaling pathway. The key features we sought to match were general behavior of the signaling components across cell types, if possible, under a given stimulus paradigm. Most of the parameters for the model listed in the Appendix and in Table 1 were based on biochemical values from our experiments (when available). More details on the choices of parameters are given in Table 1.

The reactions describing the total GRK-mediated regulation of β2AR was written out using the Law of Mass-Action considering the system to exist in equilibrium. The model was implemented using Matlab R2008 (The Mathworks, Natick, MA) and a Runge-Kutta solver. The code is available upon request. All analyses were done using Microsoft Office Excel 2003 and plots were generated using GraphPad Prism 4.

#### Simulation of perturbations of the rate constants for the various parameters

The activities or amount of individual signaling components of the pathway can vary in different cell lines or by experimental manipulations such as overexpression or knockdowns of GRKs, arrestins or phosphatases. Simulations to account for these experimental variations were performed by simply varying 2 to 50 fold the appropriate rate constants.

#### Simulation of partial agonists

The simulations for partial agonist-mediated activation of the β2AR were done by setting coupling efficiencies of each agonist relative to epinephrine (α). The coupling efficiencies/efficacies of different agonists were obtained from previous measurements [19]. The phosphorylation and internalization measurements were made at saturating concentrations for different agonists [19],[57]. Since the coupling efficiencies were set relative to epinephrine, agonist concentrations were set to saturating epinephrine concentrations (10 µM) for the simulation run.

#### Sensitivity analysis of desensitization and resensitization

The univariate sensitivity analysis for the model was carried out to test the effect of variation of individual rates on the simulation results of desensitization and resensitization. Except for ligand binding and unbinding rates all other rates were individually varied 2X, 5X, 10X, 20X above and below the measured or default rates (Table 1). The results were then plotted as the change from the average experimental measure of desensitization or resensitization at different instances of time.

### Ordinary differential equations for the GRK-mediated β2AR regulation model



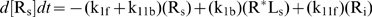
(1)

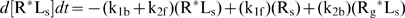
(2)

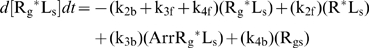
(3)

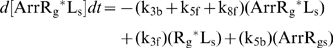
(4)

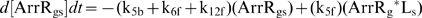
(5)

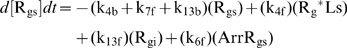
(6)

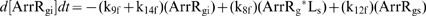
(7)

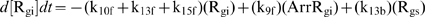
(8)

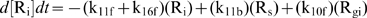
(9)

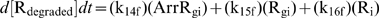
(10)Here k_xf_ and k_xb_ are the forward and backward rates for the reaction number denoted by x. R_s_ = β2AR on the plasma membrane; R^*^L_s_ = Agonist bound β2AR on the plasma membrane; R_g_^*^L_s_ = GRK phosphorylated β2AR that is on the plasma membrane and is bound to an agonist; ArrR_g_^*^L_s_ = GRK phosphorylated β2AR that is on the plasma membrane and is bound to an agonist and arrestin; ArrR_gs_ = GRK phosphorylated β2AR that is on the plasma membrane and is bound to arrestin; ArrR_gi_ = GRK phosphorylated β2AR that is in the internalized compartments and is bound to arrestin; R_gi_ = GRK phosphorylated β2AR that is in the internalized compartments; R_i_ = β2AR that is in the internalized compartments; R_degraded_ = Degraded β2AR in the cytoplasm.

The rates for models A–F shown in Figure 5 are set as follows.

Model A: All rates are set as described in Table 1, for the default model.

Model B: k_2b_ = k_7f_ = 0/min since this model disallows dephosphorylation at the plasma membrane.

Model C: k_13f_ = 0/min since this model disallows recycling of phosphorylated receptor.

Model D: k_10f_ = 0/min since this model disallows dephosphorylation of the internalized receptor.

Model E: k_10f_ = k_13f_ = 0/min since this model does not allow neither dephosphorylation of the internalized receptor nor recycling of phosphorylated receptor.

Model F: k_2b_ = k_7f_ = k_13f_ = 0/min since this model as per the currently accepted paradigm does not allow either dephosphorylation of the receptor at the plasma membrane or recycling of phosphorylated receptor.

## Supporting Information

Figure S1 Comparison of simulated time course of GRK site phosphorylation with experimentally measured phosphorylation in response to various agonists. (A) The simulated time course of GRK site phosphorylation of the β2AR in response to various agonists is compared with experimentally measured phosphorylation [19]. Phosphorylation simulations for salmeterol (α = 0.13; blue line) were done with arrestin on-rate = 0.1 k_3f_ and arrestin dissociation = 10 k_3b_ to complement with S1C-D. These results were identical to the simulations with the default rates of arrestin binding/unbinding (not shown). (B) Comparison of simulated and experimentally measured receptor phosphorylation at 2 min of agonist treatment normalized to phosphorylation achieved with 10 µM epinephrine [19]. (C) Comparison of simulated and experimentally measured receptor internalization [57] post agonist treatment. Simulated internalization for salmeterol (α = 0.13; blue lines) matches only when arrestin on-rate is 0.1 k_3f_ and arrestin dissociation is 10 k_3b_. This reduces the stability of the ArrR_g_^*^L_s_ complex a hundredfold. (D) Simulation of agonist induced receptor-desensitization.
(2.81 MB EPS)

Figure S2 Univariate sensitivity analyses of the model for desensitization. (A–Q) Desensitization of β2AR stimulation of adenylyl cyclase after treatment with 10 µM ISO simulated on twenty-fold variation of individual rates. Negative values indicate a simulated measurement higher than experimental measure.
(1.21 MB EPS)

Figure S3 Univariate sensitivity analyses of the model for resensitization. (A–Q) Resensitization of the β2AR stimulation of adenylyl cyclase post 15 min stimulation with 1µM ISO is simulated on twenty-fold variation of individual rates. Negative values indicate a simulated measurement higher than experimental measure.
(1.11 MB EPS)

Figure S4 Simulated effects of varying rates of GRK phosphorylation. Effect of variations in GRK levels or activity on phosphorylation at subsaturating concentration of ISO (50 nM) is simulated by ten-fold up or down variations in GRK phosphorylation rates.
(1.13 MB EPS)

Figure S5 Comparisons of five experimental results with simulations of model C and D. Through panels A–E we test alternate models C and D to see how well they match other experimental readouts of the β2AR signaling system besides dephosphorylation (c.f. Figure 4C, D). Comparisons of simulations (continuous lines) of model C (A–E) and model D (F–J), with experimental data obtained in HEK 293 cells stably expressing the WT β2AR (discrete data points). (A, F) Time course of GRK phosphorylation of the receptor on treatment with 50 nM ISO [19]. (B, G) Internalization of the β2AR on treatment with 1 µM ISO. Surface receptor is measured by the loss of [^3^H]CGP-12177. (C, H) Recycling of the receptor after 20 min treatment with 1µM ISO followed by rapid washout of agonist [33]. (D, I) Desensitization of β2AR stimulation of adenylyl cyclase after treatment with 10 µM ISO. (E, J) Resensitization of the β2AR stimulation of adenylyl cyclase. WT β2AR were stimulated with 1µM ISO for 15 min, followed by addition of metoprolol as described in Materials and Methods.
(1.91 MB EPS)

Figure S6 Sensitivity of simulated “Cellular Memory” to the stability of arrestin-receptor/ligand complex. Simulation of activation of β2AR by paired pulses of 1 µM ISO. Higher desensitization is obtained for the second and third pulse even on 100 fold increased stability of the arrestin-receptor/ligand complex.
(3.57 MB EPS)
